# Is Team-Based Learning Effective for the Medical Student in Difficulty?

**DOI:** 10.1007/s40670-025-02355-2

**Published:** 2025-03-12

**Authors:** Mitesh Patel

**Affiliations:** North Wales Medical School, Bangor University, College Road, Bangor, Gwynedd, North Wales LL57 2DG UK

**Keywords:** Team-based learning, Medicine, Student in difficulty, Engagement

## Abstract

Team-based learning is a widely accepted technique for small group, active learning, in medical courses. The nature of team-based learning allows students to discuss and explore topics in a structured way. The tasks that are set develop problem-solving skills and professional attitudes. Students are encouraged to collaborate and share knowledge to build confidence and a clinical language. There is more variability in the learning experience from team-based learning compared to lectures. However, for the student in difficulty, who may have failed an assessment, maintaining the current team-based learning environment may not yield beneficial results. Here, responses showed that a subset of students in difficulty do not utilise team-based learning to develop their learning. Students in difficulty can be less confident than other students and less willing to express their opinions within a group, particularly when there are highly achieving students dominating the session.

## Commentary

Medical schools use different modes of teaching to provide the best education of medical sciences possible and there have been enormous improvements to student support and mental health support in the last decade. Team-based learning is one area of development that has received praise but for the student in difficulty this environment may be challenging.

The rationale behind the use of team-based learning is to facilitate student engagement and active learning to provoke retention of material and transmission of professional attitudes [1]. To many students, the experience is largely driven by self-motivation whilst weaker students could become dependent on other members of the group. The student in difficulty may therefore be treated as burdensome by their peers. Furthermore, the competitive nature of medicine can make high-achieving students reluctant to share knowledge which further affects the team-based learning environment. There is a risk of isolation for the student in difficulty which can lead to mental health problems [11] and lack of participation and engagement in all learning activities. The issues for the student in difficulty could spiral unless there is an adequate intervention. Indeed, a large study of first year medical students has shown that students who perform poorly in team-based learning tasks have lower outcomes in their end of year examinations [3].

From the perspective of the student in difficulty, the increased demands to rectify a weak position can add significant pressure and stress to an already challenging environment. Medical students already experience higher depression, anxiety, and suicidal thoughts during the completion of the course compared to the general population and age-matched peers over the same period [4]. The challenge for the student in difficulty may be diverse and includes maintaining engagement; maintaining confidence; maintaining self-awareness; keeping on top of current learning; and maintaining a learning plan to revise material whilst also preparing for upcoming sessions. Identifying general factors that may support or hinder the student in difficulty may contribute to student performance and retention.

Interestingly, a survey of first year medical students showed that the perceived qualities to success included a committed work ethic and management of time and self, rather than medical knowledge itself [10]. Moreover, a recent review pointed towards the benefits of physical exercise to mitigate burnout and improve quality of life in medical students [12]. The results of these studies imply that self-development is an important feature of success in medicine. Consequently, it is important to consider how the student in difficulty approaches team-based learning and identify barriers.

Qualitative information was gleaned from students using a phenomenological approach of responses in a one-to-one interview. Responses were transcribed from twelve medical school students who had scored below a passing grade in a summative assessment in first- or second-year medicine. Eight were repeating.

The following questions were asked to glean how these students engaged with team-based learning tasks:

1. Could you tell me the benefits and positive aspects of your experience with team-based learning
2. Could you tell me the challenges and negative aspects of your experience with team-based learning

At the university, team-based learning followed a case-based collaborative learning approach that was learner centred but instructor led. The team-based learning sessions were a series of structured activities, including an individual readiness assurance test, team-based readiness assurance test, and short answer questions, that encouraged students to apply knowledge to real-world cases through intergroup discussions [9]. Students were encouraged to discuss questions and concepts with one another, but computers were permitted. Feedback was thereafter provided from an instructor. Each team-based learning session lasted approximately 2 h and covered one physiological system or condition. The session was delivered in line with the core elements of team-based learning, being: “team-formation,readiness assurance; immediate feedback; sequencing of in-class problem-solving; four Ss; incentive structure; and peer-review” [6].

Responses showed that students in difficulty valued team-based learning but felt that the team dynamic was not always advantageous to their learning. Both the benefits and challenges are considered below, with each of the twelve students reporting at least one benefit and one challenge during the interview.

A consistent response amongst participants was the benefit of talking to other students. Students in difficulty also stated how they preferred team-based learning to lectures as it was familiar to the classroom environment from their previous studies. Responses included:

I mostly listen to others as sometimes the lectures are too difficult to follow. The amount of material in the slides can be quite a lot so it’s a relief when I get into the group.

The social aspect kept me motivated.

Getting the answers means I can read it at home if I miss something

Sometimes we are quite confused about what we need to do. Some of the tasks can be very fun.

Talking to other students helped me to realise that I’m not alone in struggling with the workload.

I like the practice exam questions.

My focus isn’t always that good with a lecture. I like a more relaxed atmosphere.

The negative aspects to team-based learning seem to focus on the difficulty of questions and topics, and a preference of working with students who they get along with. Responses included:

The tasks that were being given are sometimes very vague and confusing, leaving me and the group unsure of what we need to do. The tutor wasn’t always clear too. I just wait for the answers to be released.

The group has some conflicting personalities which makes it quite challenging. I just work with my friend.

Sometimes, our discussions would go off track as we didn’t see the relevance of the material

We were spending time figuring out what to do.

The tutor doesn’t really give us time to discuss answers.

Some people can be annoying. I prefer working alone.

We don’t discuss the answers with each other. We split the work up and share it after.

I just google search the answers or use ChatGTP.

I don’t feel confident expressing my opinions in team-based learning. And now I try to avoid being asked questions or giving my opinions elsewhere.

Supporting the medical student in difficulty has received very little attention in the educational literature. The challenges when undertaking a medical degree traditionally include the transfer of information and acquisition of clinical skills or professional attitudes. However, for the student who has failed an assessment, the margins for error are narrow resulting in increased pressure. The approach of many medical schools is to support the student in difficulty to build a strategy for success with a revision plan. However, the dynamics of team-based learning may be the issue that needs to be resolved. Responses from students suggested that the overarching issues lay with general confidence in participating and engaging in the task. Klasen and colleagues have gone so far as to propose a workshop for medical students on dealing with failure [7]. Similarly, Koh and colleagues (2020) [8] studied the progress and results of a weaker performing team in team-based learning sessions finding that one member of this team was marginally lowering the team’s performance. However, this weaker performing students’ ability was being masked in the group and therefore the student was not receiving additional support.

Perhaps the team-based learning environment can be improved to better support students in difficulty. There is a tendency for the student in difficulty to be reserved and their teamworking skills may be affected because they have not formed a strong bond with others in the team, with those stronger taking more command. In other words, there seems to be a pattern of disillusionment in which some students can struggle to be heard in their team or even participate, waiting for answers to be released. It is perhaps telling that students in difficulty tended not to point to the value of the instructor or facilitator in their responses. Perhaps this suggests that the student in difficulty is reluctant to ask for additional support. One consideration is that the student in difficulty requires effective coaching or prompting by the instructor to stimulate their engagement.

There also seems to be a tendency to search for answers rather than discuss, or simply to wait for the answers to be released. This suggests that some students in difficulty struggle with self-directed tasks and the confidence and motivation to undertake the tasks for fear of being wrong. Alternatively, it could suggest that some students in difficulty are not prepared for collaborative techniques of learning. Team-based learning crucially depends on student engagement, understanding and ‘buy-in’ to the process [1] and that seemed to be affected in students in difficulty.

A plausible solution is to change the dynamics of team-based learning for students in difficulty by placing these students together in their own team and enabling students to regain confidence by working with a coach. By working with one another in a team-based learning environment, students in difficulty may be more likely to feel empowered to collaborate and express their opinions. Personal coaching and working on students’ confidence have been used effectively in the USA which improved stress and time management and increased self-reflection [2]. Similarly, Klasen and colleagues provided additional workshops to failing students that were supported by two surgical consultants and a life coach. The workshops enabled medical students who were failing to identify and analyse issues, gain confidence in communication, develop coping strategies, and moderate their fear of failure [7].

Wider research in this area is certainly merited given the targeted interviews of students in difficulty here. Indeed, it is worthwhile noting that medical students can view team-based learning of anatomy favourably regardless of their examination outcomes [13]. Whilst there were certain aspects of behavioural and cognitive disengagement in the responses from the students in difficulty here, there was a bigger problem with emotional disengagement. Grijpma and colleagues have suggested that medical students’ willingness to engage in classroom learning may be partly determined by the team dynamics [5]. Here, students wanted to improve and attended sessions. However, there was reluctance to verbally contribute linked to confidence and uncertainty rather than being related to a behavioural issue. By grouping students in difficulty from the results of formative assessment, early interventions including additional support and assistance for instructors could be made available. For example, instructors could make more use of open and inviting prompts, and positive communication, as suggested by Grijpma and colleagues [5]. The results of these interventions in team-based learning may have tangible benefits for students’ outcomes as noted by Carrasco and colleagues [3]. Future research could evaluate the effectiveness of these proposed interventions.

## Conclusion

Team-based learning is a tried and tested method of developing a medical student’s knowledge and professional attitudes. However, the traits that help students to gain knowledge, such as confidence in discussion, exploration of deeper thinking, and committing to an increased workload through self-directed learning, are those that the student in difficulty may try to avoid. We need to think about the way that team-based learning is delivered to better cater to students in difficulty.
